# The compensatory mechanisms for global sagittal balance in degenerative spinal kyphosis patients: a radiological analysis of muscle-skeletal associations

**DOI:** 10.1186/s12891-021-04621-x

**Published:** 2021-08-27

**Authors:** Weiwei Xia, Weiyan Wang, Zhenqi Zhu, Chenjun Liu, Shuai Xu, Fanqi Meng, Haiying Liu, Kaifeng Wang

**Affiliations:** Department of Spinal Surgery, Peking University People’s Hospital, Xizhimen south street No. 11, Xicheng district, Beijing, 100044 China

**Keywords:** Degenerative spinal kyphosis, Head position, Sagittal imbalance, Lumbar muscle degeneration

## Abstract

**Background:**

The position of the head relative to the spine can be used to evaluate the true global balance in patients with degenerative spinal kyphosis (DSK). However, it is still not clear how the position of the head is related to the spinal-pelvic parameters and lumbar muscles, which are most commonly considered.

**Methods:**

Sixty-seven patients with DSK admitted in the hospital from January 2017 to January 2019 were retrospectively analyzed. All patients had whole spine X-ray and lumbar MRI. The head position parameters include: the angles of both lines joining the center of acoustic meati (CAM) to the center of the bi-coxofemoral axis (BA) (CAM-BA) and the most superior point of dentiform apophyse of C2 odontoid (OD) to BA (OD-BA) with the vertical line; the distance between the vertical line passing CAM and the posterior upper edge of the S1 (CAM-SVA). The spinal parameters include: C7 sagittal vertical axis (C7-SVA), thoracic kyphosis (TK), thoracolumbar kyphosis (TLK), and lumbar lordosis (LL). The pelvic parameters include: pelvic incidence (PI), pelvic tilt (PT) and sacral slope (SS). The relative cross-sectional area (RCSA) of bilateral multifidus, erector spinae and psoas muscle at L3/4 and L4/5 segments were measured. The correlations between head position parameters and the spinal-pelvic parameters and RCSA of lumbar muscles were analyzed, respectively.

**Results:**

Significant positive correlations were found between each two of CAM-SVA, C7-SVA, CAM-BA and OD-BA (*p* < 0.001). SS was found to be significantly positively correlated with CAM-BA (*r* = 0.377, *p* = 0.034) and OD-BA (*r* = 0.402, *p* = 0.023). CAM-BA was found to be significantly negatively correlated with TK (*r* = − 0.367, *p* = 0.039). Significant positive correlations were found between RCSA of multifidus at L3/4 level and CAM-SVA (r = 0.413, *p* = 0.021), CAM-BA (*r* = 0.412, *p* = 0.019) and OD-BA (r = 0.366, *p* = 0.04).

**Conclusions:**

Our study showed that the head position relative to the spine were significantly correlated to some spinal-pelvic parameters, and the lower lumbar multifidus muscle. The compensatory mechanisms of the global sagittal balance status should also involve the head position area.

## Background

In the process of spinal aging, the degenerative deformity leads to a sagittal imbalance of the spine, especially for degenerative spinal kyphosis (DSK) patients. The analysis of sagittal balance is a crucial key point to optimize the management of lumbar degenerative diseases [1–3]. The traditional method evaluating the spinal sagittal balance is measuring spinal-pelvic parameters, typically determined by the C7 sagittal vertical axis (C7-SVA). Simply using C7-SVA, however, may be not enough to evaluate the sagittal balance of spine and the optimal sagittal alignment is still in controversy [4, 5]. Recent studies support the idea that the global balance should be considered in evaluating the true balance of the human body [6, 7]. Therefore, the position of the head related to the spine has been included in evaluating the true global sagittal balance, which may help to predict clinical surgery outcomes [3, 6, 7].

To maintain the postural alignment, involving the whole body from head to feet, the compensation mechanisms in head-spine-pelvis-lower limb axis contribute to keep the sagittal balance. Therefore, in order to keep the alignment of whole body’s segments (head, torso, pelvis, lower limbs), human bodies have the capacity to regulate their spinal-pelvic alignment by fine-tuning the curvature of the spine and adjusting the orientation of the pelvis [8]. The compensation mechanism is likely to make all the segments of the body correlated with each other. Therefore, the sagittal global balance is crucial for human body to be able to maintain the function of walking or standing without falling or increasing the degeneration of spine the for DSK patients. Previous study has showed the critical role of spine, pelvis and lower limbs areas in the compensatory mechanisms with severe degenerative spine, however, it is still not clear the role of the head position [9].

The atrophy of extensor muscles is correlated to a progressive kyphosis of the lumbar spine with the risk to progressively develop a global sagittal imbalance [10, 11]. Furthermore, the atrophy of back muscles has been considered to be significantly correlated with low back pain [12–14]. The incorrect head posture, e.g., flexion in the majority of time during profession, was also observed to be high risk factor related with low back pain [15]. Moreover, in our previous study, we found that the content of lean muscles at low back were correlated with spinal-pelvic parameters, which showed that the spine and the muscles could have interactions to maintain the human body sagittal balance [16]. However, it is not known whether the position of the head is correlated with back muscle degeneration in DSK patients.

Therefore, we hypothesized that the position of the head related to the spine could be correlated with other spinal-pelvic parameters and the position of the head could affect the degeneration of back muscles. The aims of this study were to:1) analyze the association between the position of the head and the spinal-pelvic alignment; 2) preliminarily explore the correlation between the position of the head and the back muscle degeneration in DSK patients.

## Methods

### Demographic characteristics

The software MedCalc (18.11.3 v) was used to calculate the sample size with Type I error at 0.05, Type II error at 0.2, difference of means was 21.4 in relative cross-sectional area (RCSA) of multifidus at L3/4 spinal level (DSK patients vs. normal controls), ratio of sample sizes in group1 /group 2 was 1. The estimated sample size was calculated to be of at least 17 participants [17].

The medical records of 67 patients who had been diagnosed with DSK when attending our hospital from January 2017 to January 2019 were retrospectively analyzed. The patients include 43 females and 24 males with age from 48 to 82 years (mean age:64.55 years). The DSK patients were diagnosed by 1) characteristic clinical features: a forward stoop with difficulty walking, adaptive postural changes in an attempt to maintain a normal standing position, such as pelvic tilt; and 2) radiographic evaluations according to Takemitsu classification using a full-length 36 in. standing lateral radiograph with degenerative changes, such as intervertebral dis narrowing, wedged or collapsed vertebral endplates, or fat infiltration of paraspinal muscles by MRI [18–20]. Imaging examination were done when the patients attended our hospital. The exclusion criteria for the study patients included: with history of tumor, tuberculosis, infection, trauma and other definite pathological changes; with history of scoliosis (cobb angle of coronal scoliosis is less than 10°), ankylosing spondylitis, and spine surgery; with neuromuscular spinal deformities caused by neurological disorders of the brain, spinal cord, and muscular system. The patients’ weight were assessed based on BMI recommended for Asians by WHO: 18.5 ~ 22.9 (normal weight), 23 ~ 24.9 (overweight), and BMI ≥ 25 (obese) [21]. The study was approved by the Medical Ethics Committee of Peking University People’s Hospital (2016PHB186–01) on November 9th 2016, and all patients gave written informed consent for their information to be stored in the hospital database and used for research. This study was conducted according to the principles expressed in the Declaration of Helsinki.

### Head-spinal-pelvic parameters

**Fig. 1:**
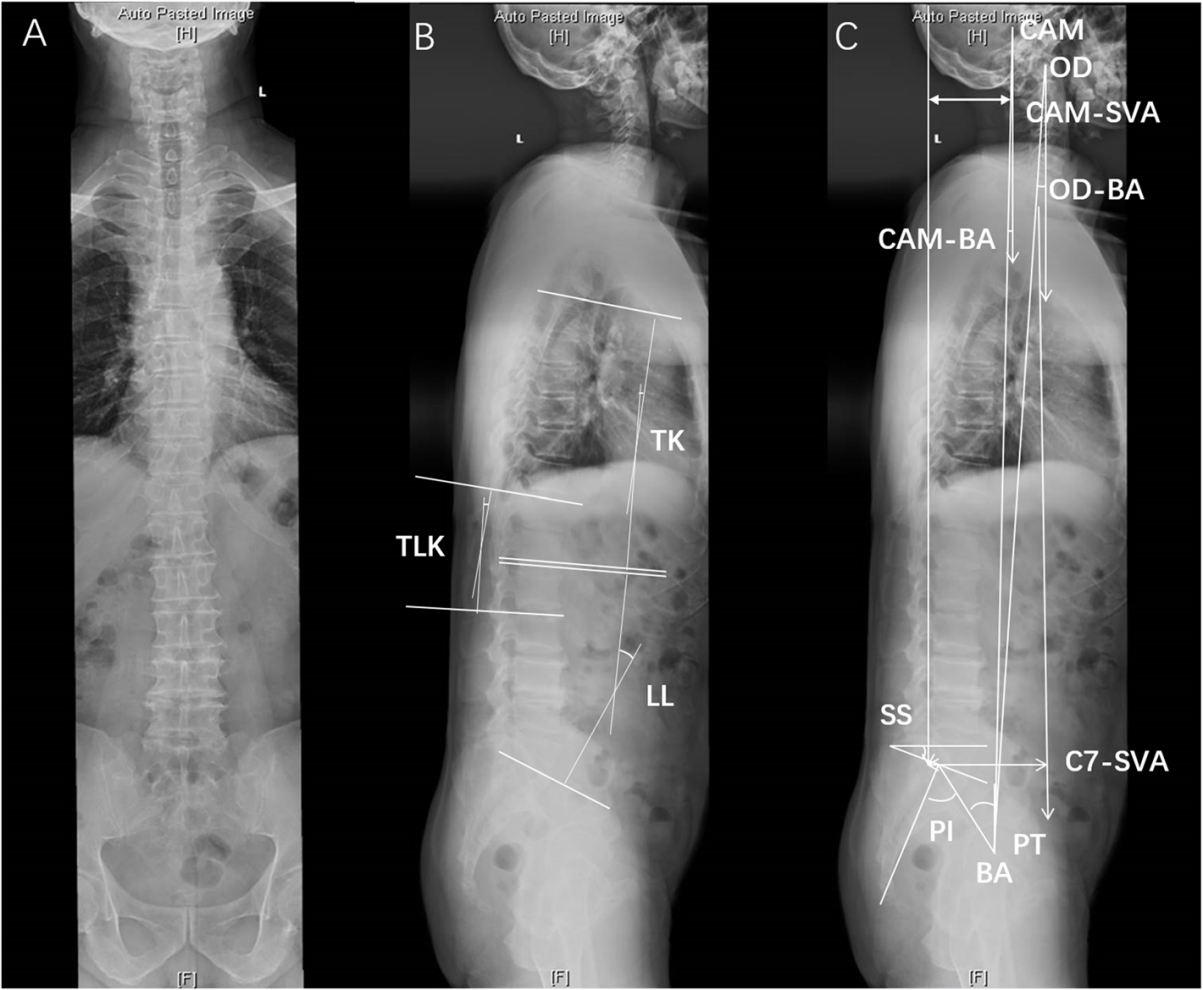
Measurements of head-spinal-pelvic parameters using the lateral full-length spine X-ray. **A** Anteroposterior x-ray; **B** and **C** Lateral x-ray. CAM: center of acoustic meati; SVA: sagittal vertical axis; BA: bicoxofemoral axis; TK: thoracic kyphosis; TLK: thoracolumbar kyphosis; LL: lumbar lordosis; PI: pelvic incidence; PT: pelvic tilt; SS: sacral slope

The position of the head relative to the spine stands for the spinal inclination. The whole spine X-ray used the standard method: all patients were asked to stand relaxedly with straightening the lower limbs and with the hands supported by two rigid poles which was shown to best assess the sagittal alignment [22]. We used the following anatomical landmarks in the lateral X-ray image for assessing head positions: the center of acoustic meati (CAM), the most superior point of dentiform apophyse of C2 odontoid (OD), the center of the bi-coxofemoral axis (BA). The measurements include: angles of both lines joining CAM to BA (CAM-BA) and OD to BA (OD-BA) with the vertical line; distance between the sagittal vertical axis (SVA) and the center of gravity of the head, i.e., SVA-CAM. The spinal parameters include: C7 sagittal vertical axis (C7-SVA), thoracic kyphosis (TK), thoracolumbar kyphosis (TLK), and lumbar lordosis (LL). The pelvic parameters include: pelvic incidence (PI), pelvic tilt (PT) and sacral slope (SS) [23](Fig. 1). The degrees of lordosis was defined positive and kyphosis was defined negative [16]. If the line of CAM-BA or OD-BA was at the left side of the vertical line, the degrees were positive; if the line of CAM-BA or OD-BA was at the right side of the vertical line, the degrees were negative.

### Quantitative muscle measurement

**Fig. 2:**
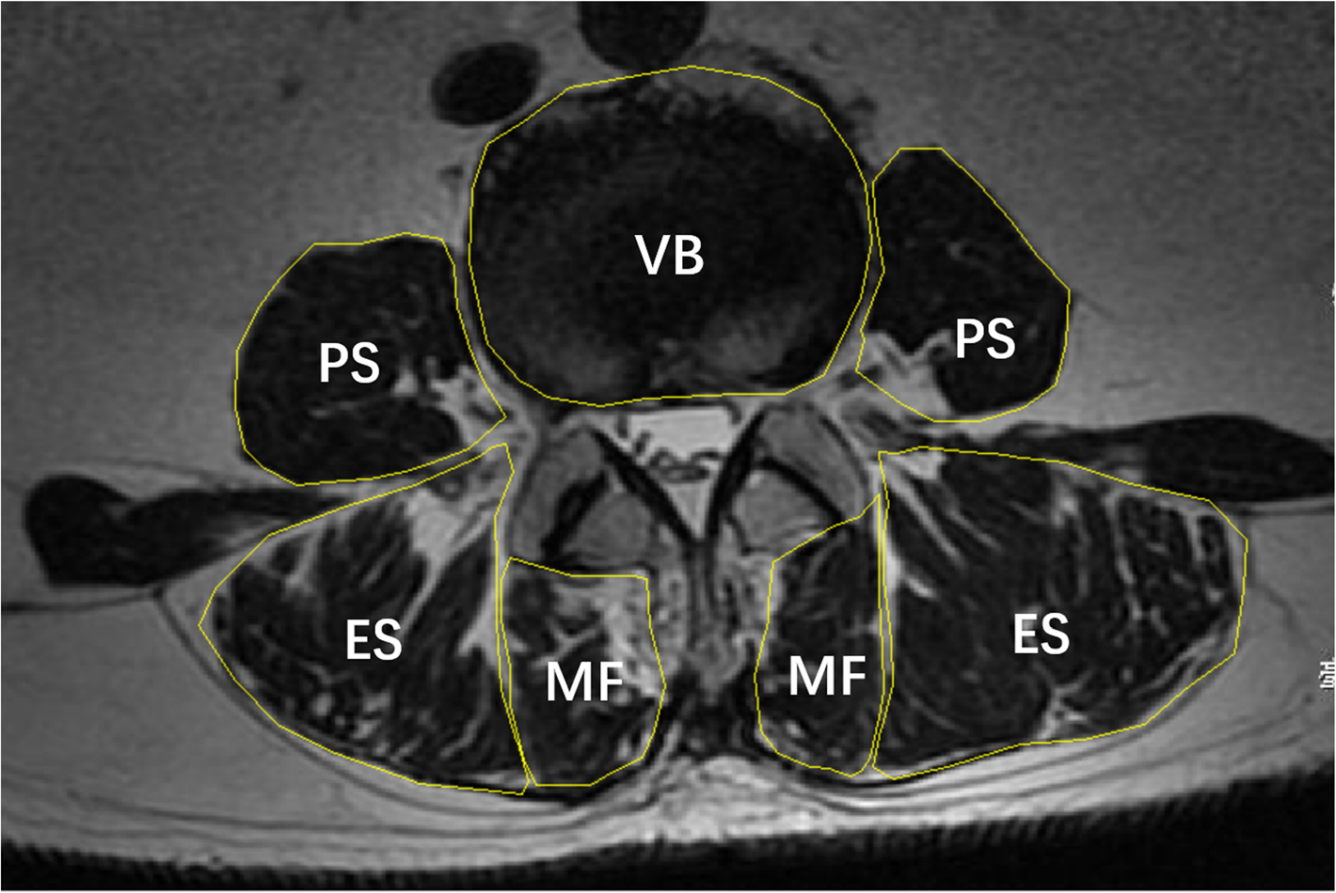
Axial T2-weighted MR image demonstrating measurements of RCSA of different muscle groups and the VB by creating ROIs. PS = psoas muscle, ES = erector spinae, MF = multifidus, VB = vertebrae body

The T2-weighted MRI image data were acquired on the 1.5 T Sigma whole body imaging system (General Electric Medical Systems, WI). Scans used a T2-weighted fast spin echo sequence, with TR/TE times of 4274/107 ms, slice thickness of 4 mm, acquisition matrix 224 × 320, pixel bandwidth 195 Hz/Px, and echo train length 17. The patients were placed in the supine position, with their legs straight and the lumbar spine in a neutral posture. Measurements were obtained from a single slice at the inferior border of each disc, from L3/4 to L4/5, which were obtained parallel to the superior endplate of the lower vertebra at each level. Six regions of interest (ROI) for the muscles were manually defined per slice: the ROI for the multifidus, the erector spinae and the psoas muscle were defined bilaterally [24] (Fig. 2). The method of defining the ROIs of each muscle is as followings: the medial border of multifidus is most superficial aspect of the spinous process, the anterior border follows the lamina, mammillary process, zygapophyseal joint, the lateral border follows the fascial line between erector spinae, the posterior border extends the epimysium of multifidus; the medial border of erector spinae is the fascial line between multifidus, the anterior border runs along the transverse the process, the lateral border follows the rounded contour of the fascial boundary surrounding iliocostalis, the posterior border follows the aponeurosis of erector spinae; the border of psoas muscle follows the surrounding aponeurosis of psoas [24]. The muscle measurements include total cross-sectional area (CSA) (i.e., muscle size) and functional CSA (FCSA, i.e., lean muscle). The FCSA was estimated according to the method proposed by Ranson et al. [25], with a threshold range from 0 to 120 for the gray scale to only include those pixels representing lean muscle content from each muscle CSA. This method has also been used for calculating the fat infiltration (FI) [26]. Muscle CSA was determined by constructing the border of each muscle using polygon tool in the ImageJ software (version 1.52, National Institutes of Health, USA). To compensate for the bias caused by the relative body size of the individual on muscle CSA, we calculated the relative CSA (RCSA), i.e., dividing the muscle FCSA by the CSA of the superior endplate of the lower vertebrae at each spinal level. RCSA was used to evaluate the lumbar muscularity to stabilize the spine column. FI was calculated as follows: FI = (CSA-FCSA)/CSA. Each image was assessed two times, and the average value was calculated as the final result. The reliability of RCSA was analyzed by computing the intra-rater and inter-rater ICC values. The results were: intra-rater ICC of RCSA for multifidus, erector spinae and psoas were 0.987 (95%CI: 0.968–0.995), 0.999 (95%CI: 0.997–0.999), and 1 (95%CI: 0.999,1); inter-rater ICC of RCSA for multifidus, erector spinae and psoas were 0.965 (95%CI: 0.911,0.986), 0.997 (95%CI: 0.991,0.999), and 0.997 (95%CI: 0.992, 0.999). According to the ICC values, the measurements showed excellent reliability.

### Data evaluation and statistical analysis

Statistical Package for Social Science (SPSS, v23.0, Chicago, IL, USA) software was used for data analysis. Data were tested for normality by Kolmogorov–Smirnov test which presented a normal distribution. The correlation between head position parameters and individual muscle degeneration at each lumbar spinal level and the correlation between head position parameters and spinal-pelvic parameters was analyzed by Pearson correlation analysis. The Pearson correlation analysis was also used to determine the relationships between each two of the head position parameters and C7-SVA (i.e., CAM-SVA, CAM-BA, OD-BA, and C7-SVA). FI and RCSA of multifidus, erector spinae and psoas muscle were compared at L3/4 and L4/5 spinal level, respectively. In addition, FI and RCSA of the three muscles were compared between L3/4 and L4/5. The correlations between age and the above measurements were assessed by Pearson correlation analysis. The comparisons used one-way ANOVA with multiple comparisons in paired t-test. The data is presented as mean values±SD (standard deviation of the mean). *P*-value< 0.05 was considered to be statistically significant.

## Results

Sixty-seven DSK patients (43 females) with age ranging from 48 to 82 years (age = 64.5 ± 8.3 years, mean ± SD) with complete image data were included in this study. According to Takemitsu classification method for degenerative kyphosis, the patients included 22 cases of type I (32.8%), 30 cases of type II (44.8%), 14 cases of type III (20.9%), 1 case of type IV (1.5%) [18]. According to the BMI classification, 6 patients (9%) were in normal weight, 16 patients (23.9%) were overweight, 35 patients (52.5%) were obese [21].

The results of measurements include CAM-SVA (94.89 ± 49.47 mm), C7-SVA (68.09 ± 47.6 mm), CAM-BA (2.73 ± 3.49 °), OD-BA (1.99 ± 3.65°), TK (− 22.84 ± 11.47°), TLK (− 8.52 ± 9.62 °), LL (25.75 ± 11.54°), PI (45.54 ± 8.99°), PT (21.28 ± 8.09°), SS (24.26 ± 8.58°). Significant correlations were found between each two of CAM-SVA, C7-SVA, CAM-BA and OD-BA, which are shown in Table 1. CAM-BA was found to be correlated to TK (*r* = − 0.367, F = 4.664, *p* = 0.039) and SS (r = 0.377, F = 4.962, *p* = 0.034). OD-BA was found to be correlated to SS (*r* = 0.402, F = 5.788, *p* = 0.023) (Table 2).

**Table 1:** Correlations between head position parameters and C7-SVA

	CAM-SVA	CAM-BA	OD-BA	C7-SVA
CAM-SVA	–	–	–	–
CAM-BA	*r* = 0.918, *p* < 0.001	–	–	–
OD-BA	*r* = 0.897, *p* < 0.001	*r* = 0.976, *p* < 0.001	–	–
C7-SVA	*r* = 0.819, *p* < 0.001	*r* = 0.74, *p* < 0.001	*r* = 0.784, *p* < 0.001	–

*C7-SVA* C7 sagittal vertical axis, *CAM-SVA* Center of acoustic meati-Sagittal vertical axis, *CAM-BA* Center of acoustic meati-Bicoxofemoral axis, *OD-BA* Odontoid- Bicoxofemoral axis

**Table 2:** Correlations between head position and spinal-pelvic parameters (r, p)

	TK	TLK	LL	PI	PT	SS
CAM-SVA	−0.219,0.229	−0.186,0.308	− 0.329,0.066	0.317,0.077	0.167,0.36	0.174,0.341
CAM-BA	−0.367,0.039*	−0.261,0.149	− 0.161,0.379	0.228,0.209	− 0.146,0.425	0.377,0.034*
OD-BA	−0.295,0.101	−0.267,0.139	− 0.166,0.364	0.249,0.169	− 0.15,0.414	0.402,0.023*

*CAM-SVA* Center of acoustic meati-Sagittal vertical axis, *CAM-BA* Center of acoustic meati-Bicoxofemoral axis, *OD-BA* Odontoid-Bicoxofemoral axis, *TK* Thoracic kyphosis, *TLK* Thoracolumbar kyphosis, *LL* Lumbar lordosis, *PI* Pelvic incidence, *PT* Pelvic tilt, *SS* Sacral slope. *: *p* < 0.05

At L3/4 level, the FI of multifidus was 12.9 ± 6.79% (mean ± SD), the FI of erector spinae was 12.59 ± 6.77%, the FI of psoas was 4.06 ± 4.4%; At L4/5 level, the FI of multifidus was 16.49 ± 6.5% (mean ± SD), the FI of erector spinae was 17.54 ± 9.14%, the FI of psoas was 2.12 ± 2.74%. At L3/4 level, the RCSA of multifidus was 42.92 ± 15.1% (mean ± SD), the RCSA of erector spinae was 127.91 ± 33.27%, the RCSA of psoas was 82.5 ± 23.6%; At L4/5 level, the RCSA of multifidus was 58.77 ± 13.63% (mean ± SD), the RCSA of erector spinae was 97.55 ± 24.11%, the RCSA of psoas was 102.35 ± 27.16%.

**Fig. 3:**
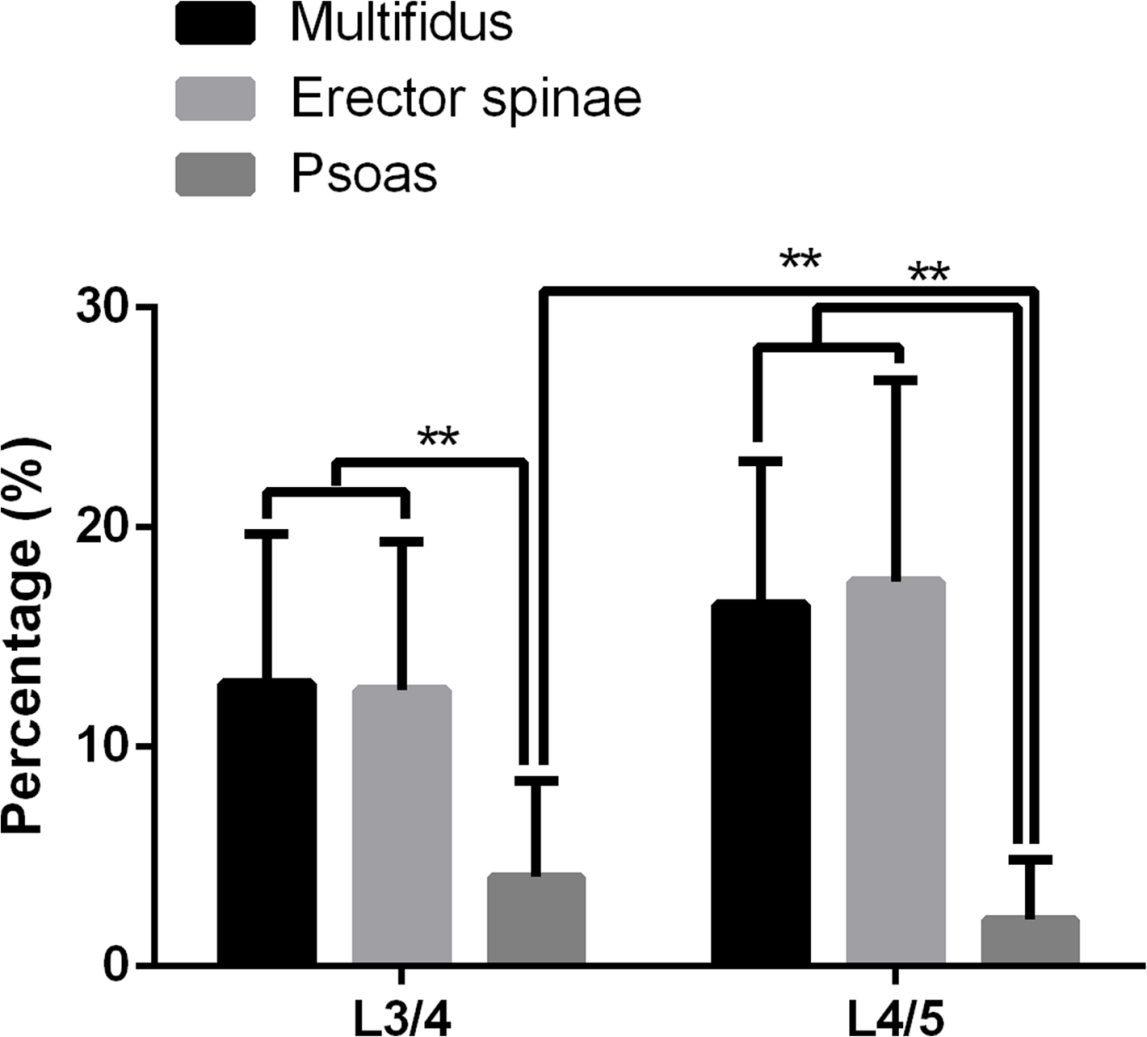
Fat infiltration (FI) of multifidus, erector spinae and psoas at each spinal level from L3/4 to L4/5. **: *p* < 0.01

**Fig. 4:**
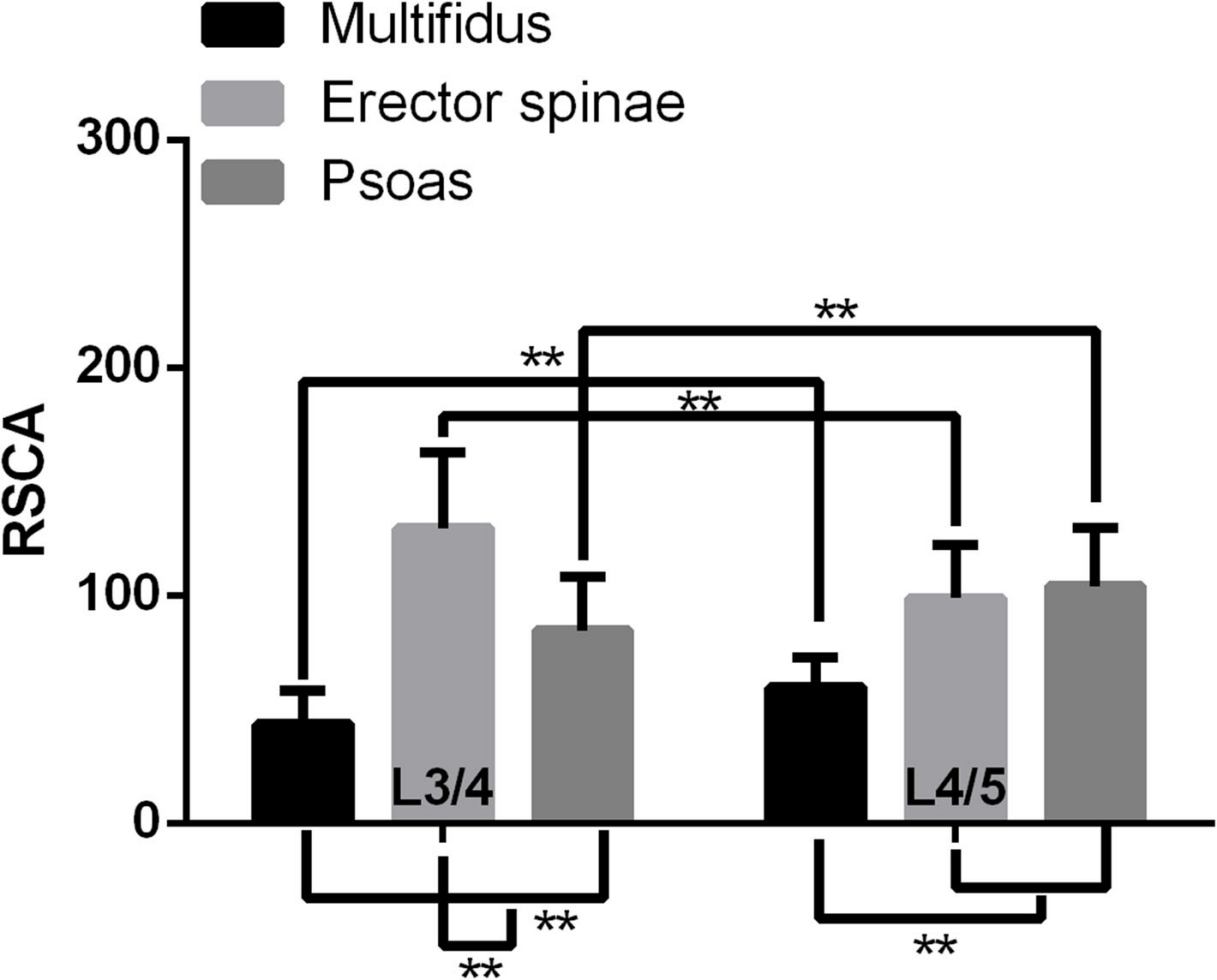
Relative cross-section area (RCSA) of multifidus, erector spinae and psoas at each spinal level from L3/4 to L4/5. **: *p* < 0.01

The FI of multifidus and erector spinae at L3/4 were not found to be different from L4/5. The FI of psoas at L3/4 was found to be higher than L4/5 (*p* < 0.01). The FI of multifidus and erector spinae were higher than that of psoas at L3/4 and L4/5 levels (*p* < 0.01) (Fig. 3). The relative cross-sectional area (RCSA) of multifidus and psoas at L3/4 were found to be lower than L4/5 (*p* < 0.01). The RCSA of erector spinae at L3/4 was found to be higher than L4/5 (*p* < 0.01). At L3/4 spinal level, the RCSA of erector spinae was higher than multifidus and psoas (*p* < 0.01), the RCSA of psoas was higher than multifidus (*p* < 0.01). At L4/5, the RCSA of erector spinae and psoas were higher than that of multifidus (*p* < 0.01) (Fig. 4).

The RCSA of multifidus at L3/4 level was found to be correlated to CAM-SVA (*r* = 0.413, F = 5.965, *p* = 0.021), CAM-BA (*r* = 0.412, F = 6.131, *p* = 0.019) and OD-BA (*r* = 0.366, F = 4.635, *p* = 0.04) (Table. 3). Age was found to be negatively correlated to the RCSA of multifidus (*r* = − 0.507, F = 10.04 *p* = 0.004) and psoas (*r* = − 0.437, F = 6.854, *p* = 0.014) muscles (i.e., mean RCSA of muscle at L3/4 and L4/5).

**Table 3:** Correlations between head position and RCSA of muscles (r, p)

	Multifidus	Erector spinae	Psoas
CAM-SVA	0.413,0.021*(L3/4) −0.02,0.914(L4/5)	0.038,0.835(L3/4) 0.194,0.288(L4/5)	0.04,0.827(L3/4) 0.181,0.323(L4/5)
CAM-BA	0.412,0.019*(L3/4) 0.124,0.5(L4/5)	−0.028,0.879(L3/4) 0.268,0.139(L4/5)	0.035,0.850(L3/4) 0.138,0.452(L4/5)
OD-BA	0.366,0.039*(L3/4) 0.126,0.493(L4/5)	0.005,0.978(L3/4) 0.313,0.081(L4/5)	0.033,0.857(L3/4) 0.15,0.413(L4/5)

*CAM-SVA* Center of acoustic meati-Sagittal vertical axis, *CAM-BA* Center of acoustic meati-Bicoxofemoral axis, *OD-BA* Odontoid-Bicoxofemoral axis. *: *p* < 0.05

## Discussion

The evaluation of the global balance including head, spine and pelvis, even the lower limbs, is very important for postponing the development of related degenerative spinal diseases or deformities and helping to reduce the occurrence of postoperative complications. However, the position of the head relative to the trunk is not well known. In the present study, we found that the position of the head is critically correlated with certain spinal-pelvic parameters and the muscularity of multifidus at L3/4 level was found to be significantly positively correlated with the anteversion of the head.

It was reported that the inclination of the head compared to the pelvis could be a good indication of the postural trouble [7, 8, 27]. According to a 3-D construction study from Amabile et al., the inclination of the line joining CAM and HA (the middle of both centers of femoral heads (hip axis, HA)) and the line joining OD and HA are the less variable among subjects (SD = 2°) [28]. However, in the present study, the SD for CAM-BA and OD-BA was 3.49° and 3.65°, respectively, which were higher than that study. This may due to:1) Our study used the 2-D X-ray to join the line between CAM and BA, but not a 3-D construction image joining CAM and HA. This may cause certain variations. 2) Most of the patients in our study were old degenerative kyphosis patients from type I to type III according to Takemitsu classification, whereas they used asymptomatic adults. This may cause larger variations of the parameters in older patients. The average values of CAM-BA and OD-BA were 2.73° and 1.99°, respectively. These are lower than the results (CAM-HA and OD-HA were 3° in average) of Amabile et al.’s study [28]. This may because that most of the patients in our study were flat back patients with loss of thoracic kyphosis and lumbar lordosis. This leads to a decrease of head anteversion which could reduce the angles of CAM-HA and OD-HA. In our study, each two of CAM-SVA, CAM-BA, C7-SVA, OD-BA were correlated with each other. Therefore, these are complementary parameters in describing the inclination of cervical spine and head relative to the trunk. The C7 point can be useful when CAM and/or OD are not visible on the radiographies [8]. Age was not found to be significantly correlated with head position parameters, which is in agreement with the previous study in asymptomatic old adults [28]. This indicated that compared to the degeneration of spinal column and paraspinal muscles, age may be not a direct factor to affect the head position.

In the present study, we found that CAM-BA was correlated with TK, which showed that a higher angle of CAM-BA was usually accompanied by a higher angle of TK. This is consistent with other studies: a forward head posture was shown to be accompanied by a relatively more flexed lower cervical spine [29]; namely, the cervical kyphosis deformities usually have the thoracal kyphosis [6, 30]. This is a common scene that a severer anteversion of the head is commonly accompanied with a larger thoracic kyphosis, e.g., stoop [29, 30]. CAM-BA and OD-BA were found to be positively correlated with SS, which may due to the compensatory mechanisms between the position of head and the rotation of sacrum. From the present study, we can see that the rotation of sacrum could compensate for the anteversion of the head to maintain the sagittal balance. The possible reason for just finding the positive correlation between CAM-BA, OD-BA and SS, but not between PT is that the sacrum is part of spine which directly connects with the head, so the rotation of sacrum may more closely correlate with the position of head [31]. Other studies also suggested the positive correlation between thoracic kyphosis and sacrum inclination; thereafter, the correlation found between the head forward inclination (CAM-BA) and SS could be assumed due to the correlation between CAM-BA and TK which was found in our study [18, 32]. From the results, we can see that the head-spinal-pelvic parameters are inter-related. The global sagittal balance of the DSK patients should be assessed comprehensively.

Muscles have an important role for maintaining the balance of posture, and lumbar muscles are important for maintaining the stability of lumbar segments [33, 34]. Therefore, the dysfunction of paraspinal muscles at lumbar region can aggravate spinal deformity, resulting in the sagittal and/or coronal spinal imbalance. Atrophy of extensor muscles resulting in a progressive kyphosis of the lumbar spine with the risk to progressively develop a global sagittal imbalance [11, 35]. Previous studies have shown that the degeneration of paraspinal muscles, i.e., fat infiltration, in patients with degenerative spinal deformities was severer compared to normal control subjects [10, 36]. Paraspinal muscles at L3/4 and L4/5 levels were chosen in our study because the CSA of multifidus, erector spinae and psoas are all relatively large on average. The FI and RCSA showed differences among different muscles and also between different spinal levels in our study, which further confirms that different muscles at different spinal levels have different roles in keeping human postures and balance [16]. Erector spinae is considered to have a greater role in producing spinal movement, but multifidus is considered responsible for small movements to stabilize the spine and maintain the lumbar curvature [37, 38]. The erector spinae is situated more superficially and spans larger sections of the spine, whereas the multifidus muscle is located deeply, attaching to the lumbar vertebrae in sections [37, 38]. Although, we found that the RCSA of multifidus was lower than erector spinae and psoas at L3/4 and the RCSA of multifidus at L3/4 was lower than L4/5, in our study, the RCSA of multifidus at L3/4 level was found to be correlated to CAM-SVA, CAM-BA and OD-BA. This means that a more anteversion of the head needs a more muscularity of multifidus at L3/4 level in the compensatory point of view. The apical vertebra of the lumbar spinal lordosis is usually L3 or L4 vertebra. As a result, the multifidus located at L3/L4 level may need more muscularity to maintain the lumbar lordosis when the head anteverts in a higher angle. No significant correlation was found between RCSA of erector spinae and psoas. Erector spinae is a large muscle running the length of the vertebral column, so there may be other parts at specific spinal level instead of L3/4 to compensate the anteversion of the head. Psoas muscle is primarily a hip flexor which may contribute less to compensate the anteversion of the head [39]. What we get more from this study is that the long time of head anteversion might cause low back pain, as this posture could lead to a sustained muscle tension at low back.

The analysis of sagittal balance is critically important to optimize the management of lumbar degenerative diseases, especially when spinal instrumentation is intended [2, 40]. It was reported that the cervical inclination angle (CIA) and along with the OD-HA, which describes the adequacy of the global balance in young and elderly asymptomatic populations, are two important parameters that could affect junctional breakdowns in thoraco-lumbar fusion surgeries [41]. The preoperative sagittal malalignment or imbalance showed a significant relationship with incidence of proximal junctional kyphosis (PJK). It was reported that the preoperative C7-SVA demonstrated a significant relationship with incidence of PJK [42]. The weight of the head in different inclination angles can cause different torque on spinal column, which may lead to different degree of injuries to the spine [41]. In our study, there were close relations between the position of the head and the spinal-pelvic alignment and back muscles indicating the underlying compensated mechanisms [3, 6]. The position of the head relative to the spine should be taken more consideration when performing spinal correction surgeries.

There are also several limitations in our study: Firstly, this study may have been limited by the small number of patients. The scarcity of degenerative kyphosis deformity patients who underwent MRI of the lumbar spine and full-length spine X-ray examination was the main causative factor. A large population based multicenter investigation will be more meaningful and help to clarify the compensatory mechanisms in the head-spine-pelvis system. Secondly, a control group of normal healthy subjects which was lacked in our study would help to observe the head inclination in DSK patients. Thirdly, this study still lacks the measurements of lower limbs. Further studies, including the lower limbs parameters, e.g., EOS imaging measurements capturing head-to-toe images, will be required to clarify these issues [43].

## Conclusions

Our study showed that the head position relative to the spine were significantly correlated to some spinal-pelvic parameters, and the lower lumbar multifidus muscle. The results of this study suggest the existence of muscle-skeletal associations at sagittal plane in DSK patients. The compensatory mechanisms indicated in head-spine-pelvis axis system should play an important part in maintaining the human body global balance. Our study provides the image modalities of DSK patients which can give suggestions for the treatment for DSK patients. We hope that our data would stimulate further study about the effect of head position on the surgical treatment of degenerative sagittal spinal deformities.

## Data Availability

The datasets used and/or analyzed during the current study are available from the corresponding author on reasonable request.

## Abbreviations

DSK: Degenerative spinal kyphosis
C7-SVA: C7 sagittal vertical axis
CAM-SVA: Center of acoustic meati-Sagittal vertical axis
CAM-BA: Center of acoustic meati-Bicoxofemoral axis
OD-BA: Odontoid-Bicoxofemoral axis
TK: Thoracic kyphosis
TLK: Thoracolumbar kyphosis
LL: Lumbar lordosis
PI: Pelvic incidence
PT: Pelvic tilt
SS: Sacral slope
CSA: Cross-sectional area
FCSA: Functional cross-sectional area
RCSA: Relative cross-sectional area
FI: Fat infiltration
PJK: Proximal junctional kyphosis
MRI: Magnetic resonance imaging
